# Subcutaneous Granuloma Annulare Mimicking Rheumatoid Nodules: A Diagnostic Challenge

**DOI:** 10.7759/cureus.107187

**Published:** 2026-04-16

**Authors:** Meghana Vankina, Srilatha Kothandaraman

**Affiliations:** 1 Internal Medicine, Tulane University School of Medicine, New Orleans, USA; 2 Rheumatology, Texas A&M College of Medicine, Bryan, USA; 3 Rheumatology, Baylor Scott & White Health, Dallas, USA

**Keywords:** autoimmune markers, histopathology, palisaded granulomatous dermatitis, rheumatoid nodules, subcutaneous granuloma annulare

## Abstract

Subcutaneous granuloma annulare (SGA) is a rare skin condition that has overlapping features with rheumatoid nodules due to similar clinical and histological features. A 42-year-old female with a history of celiac disease and a family history of rheumatoid arthritis developed progressively painful nodules on her right hand. She was initially thought to have rheumatoid arthritis, but her serological markers were negative, and an incisional biopsy revealed palisaded granulomas with central mucin and necrobiotic collagen that led to a diagnosis of SGA. Differentiating between these two conditions is important because rheumatoid nodules require immunosuppressive therapy due to systemic inflammation, while SGA is a self-limiting benign condition. This case highlights the need for a biopsy of atypical subcutaneous nodules and the importance of combining clinical, pathological, and interdisciplinary expertise to ensure accurate diagnosis and the best patient care.

## Introduction

Subcutaneous granuloma annulare (SGA) is a rare variant of granuloma annulare characterized by deep dermal or subcutaneous nodules. It is more commonly described in children but can also occur in adults, where it often presents in periarticular areas such as the hands and lower extremities. SGA can closely mimic rheumatoid nodules both clinically and histologically, creating a diagnostic challenge [1].

Distinguishing between these entities is essential due to differences in management and prognosis. Rheumatoid nodules are associated with systemic inflammatory disease and may require immunosuppressive therapy, whereas SGA is a benign, self-limiting condition that is often managed conservatively. Histopathologically, both conditions demonstrate palisading granulomas; however, SGA is characterized by central mucin and deposition and necrobiotic collagen, while rheumatoid nodules exhibit fibrinoid necrosis with peripheral fibrosis. We present a case of SGA mimicking rheumatoid nodules, highlighting the importance of clinicopathologic correlation in obtaining an accurate diagnosis.

## Case presentation

A 42-year-old right-handed female with a history of celiac disease managed with a gluten-free diet and a family history of rheumatoid arthritis in her father presented with progressively painful nodules on the digits of her right hand. She was referred by her dermatologist with a presumed diagnosis of rheumatoid arthritis. For over a year, the nodules had been increasing in size, causing functional limitations such as gripping objects and cosmetic concerns.

**Figure 1:**
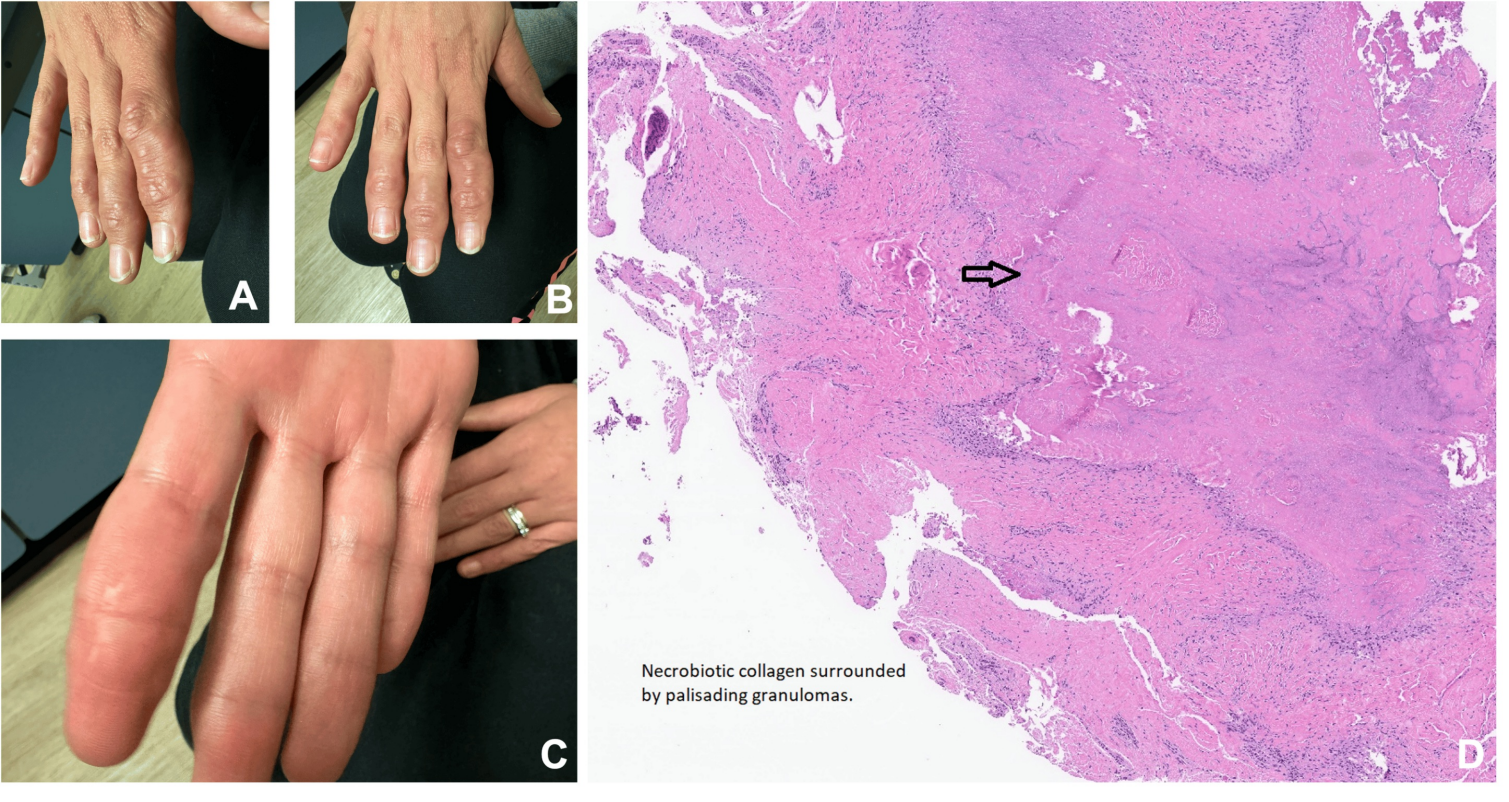
Clinical examination findings of subcutaneous nodules in the right hand (A) Multiple firm subcutaneous nodules over the right second distal interphalangeal (DIP) and proximal interphalangeal (PIP) joints. (B) Additional nodules on the right third DIP, right fourth DIP, and between the PIP and DIP joints of the second and third fingers. (C) A prominent palmar nodule on the right second finger. (D) Histopathology of the biopsied nodule shows palisading granulomas with central mucin and necrobiotic collagen.

On examination, multiple firm, subcutaneous nodules were noted on her right hand. These included lesions over the second distal interphalangeal (DIP) and proximal interphalangeal (PIP) joints (Figure 1A), the right third DIP, the right fourth DIP, and areas between the PIP and DIP joints of the second and third fingers (Figure 1B). A notable nodule on the palmar side of her right second finger was also observed (Figure 1C). There was no overlying erythema or warmth over the nodules.

Laboratory results showed a negative rheumatoid factor, negative anti-cyclic citrullinated peptide antibodies, and other negative serological markers. X-ray imaging of the hand revealed no abnormalities. Despite her family history of rheumatoid arthritis, the patient had no clinical symptoms such as joint pain, morning stiffness, or synovitis.

Due to the absence of systemic inflammatory signs or serological findings, a biopsy was pursued. An incisional biopsy of the nodule revealed palisaded granulomas with central mucin and necrobiotic collagen (Figure 1D), consistent with subcutaneous granuloma annulare (SGA). Features typically seen in rheumatoid nodules, such as central fibrinoid necrosis and peripheral fibrosis, were absent.

After integrating clinical findings, pathology results, and interdisciplinary input, a final diagnosis of subcutaneous granuloma annulare was established. The patient was reassured about the benign nature of her condition and was counseled on conservative management strategies.

## Discussion

Subcutaneous granuloma annulare is a rare form of granuloma annulare and is distinguished by the presence of palpable subcutaneous nodules [1,2]. SGA is more common in children but can occur in adults, where it tends to present in juxta-articular areas such as the hands, lower legs, head, and buttocks. Although its exact pathogenesis is unknown, SGA is thought to develop through a delayed-type hypersensitivity reaction that causes connective tissue breakdown [3]. Rheumatoid nodules are associated with known rheumatoid arthritis, occur at pressure points, and often indicate more severe disease. Rheumatoid nodules require immunosuppressive therapy, whereas SGA is generally benign and self-limiting [2].

The distinction between SGA and rheumatoid nodules can be achieved through histopathological examination. While both conditions demonstrate palisaded granulomas, SGA is characterized by central mucin deposition with necrobiotic collagen, whereas rheumatoid nodules exhibit fibrinoid necrosis with peripheral fibrosis [1,4]. In atypical presentations, such as in our patient who had no synovitis and negative serologies, clinical assessment alone may be insufficient. In such cases, an early biopsy is crucial for a definitive diagnosis.

When evaluating subcutaneous nodules, several other differential diagnoses should be considered, such as necrobiosis lipoidica, epithelioid sarcoma, dermoid cysts, and tendinous xanthomas [2]. Necrobiosis lipoidica presents as atrophic yellowish plaques, primarily on the lower extremities, but histologically contains layered zones of collagen degeneration, lipids, and plasma cells [4,5]. Epithelioid sarcoma is a rare but aggressive soft tissue tumor that may resemble granulomatous inflammation histologically and should be suspected when lesions are persistent, progressively enlarging, or ulcerative [6]. Dermoid cysts are benign congenital lesions that can present as subcutaneous nodules but are differentiated by their cystic nature and the presence of ectodermal components such as hair [7]. Tendinous xanthomas appear as firm, painless nodules over tendons and are linked to hyperlipidemia. Given the overlap in clinical and histological features among the different differentials, a multidisciplinary approach involving dermatologists, rheumatologists, and pathologists can aid in making an accurate diagnosis.

The management of SGA is generally conservative, as many cases resolve spontaneously over time. For asymptomatic patients, observation is the primary approach, though the resolution process may take several months [2]. For patients who have symptomatic or persistent nodules, intralesional corticosteroid therapy is often preferred over non-surgical intervention. Numerous studies have demonstrated that injecting triamcinolone acetonide at a concentration of 10-20 mg/mL every 4-6 weeks results in successful reduction of lesion size for most patients [2]. Topical corticosteroids are minimally effective due to their limited ability to penetrate dermal and subcutaneous tissue [2].

For patients with multiple or treatment-resistant lesions, systemic therapy may be considered, although data supporting its use in SGA is limited. The antimalarial agent hydroxychloroquine is used to treat refractory granuloma annulare variants in dosages of 200 to 400 mg per day [8]. Patients who fail antimalarial therapy can take methotrexate at low doses (7.5-15 mg weekly) as an alternative treatment [9]. Oral corticosteroids have shown mixed results; some cases have resolved completely following a short course of prednisone, while others have only experienced partial improvement [2]. Systemic therapy is generally reserved for cases where lesions significantly impact function or quality of life. Lastly, surgical excision provides quick results, but some patients may experience regrowth of the lesions [2].

The efficacy of topical calcineurin inhibitors such as tacrolimus and pimecrolimus remains inconsistent when used as treatment options. Intralesional methotrexate has been used in some refractory cases, but its effectiveness for SGA remains undefined. TNF-α inhibitors have shown effectiveness in generalized granuloma annulare cases, although their use for SGA requires further investigation because they are used off-label [10]. Phototherapy and narrowband UVB have shown promise in treating generalized granuloma annulare but have not been widely studied in SGA [11]. The rarity of SGA prevents researchers from conducting extensive clinical trials, which means treatment decisions depend on case reports and small retrospective studies.

Increased awareness of SGA can help distinguish it from clinically similar conditions, reducing the risk of misdiagnosis and unnecessary treatment. This case highlights the importance of a biopsy in atypical presentations of subcutaneous nodules, as early pathological assessment can aid in making an accurate diagnosis. While more research is needed to standardize treatment, recognizing SGA as a benign, self-limiting condition is important for proper management and better patient outcomes. This case emphasizes that early biopsy in atypical presentations can prevent misdiagnosis and unnecessary immunosuppressive therapy.

## Conclusions

This case highlights the importance of distinguishing subcutaneous granuloma annulare from rheumatoid nodules to avoid unnecessary treatment. Given the overlap in clinical and histopathological features, a comprehensive diagnostic approach incorporating clinical correlation, serologic evaluation, and dermatopathologic review is essential. Although this represents a single case, it emphasizes the value of early biopsy in atypical presentations and the importance of clinicopathologic correlation in guiding appropriate management. 
